# Microstructural White Matter Characteristics in Parkinson's Disease With Depression: A Diffusion Tensor Imaging Replication Study

**DOI:** 10.3389/fneur.2019.00884

**Published:** 2019-08-13

**Authors:** Colleen Lacey, Lisa Ohlhauser, Jodie Reanna Gawryluk

**Affiliations:** ^1^Psychology Department, University of Victoria, Victoria, BC, Canada; ^2^The Institute on Aging and Lifelong Health, University of Victoria, Victoria, BC, Canada; ^3^The Division of Medical Sciences, University of Victoria, Victoria, BC, Canada

**Keywords:** Parkinson's disease, depression, diffusion tensor imaging, white matter, replication

## Abstract

**Background:** Clarifying the neuropathology of depression as a symptom of Parkinson's disease (PD) has been the goal of recent neuroimaging studies; however, results have been conflicting and lack replication. The purpose of the current study was to replicate recent methods that have used diffusion tensor imaging (DTI) to compare individuals with PD with and without depression and to extend previous findings to allow for a better understanding of the results.

**Methods:** Thirty-seven participants with *de novo* PD were retrieved from the Parkinson's Progression Marker's Initiative (PPMI) and were separated into a depressed PD group (dPD) or a non-depressed PD group (ndPD). Groups were determined based on scores on the Geriatric Depression Scale Short Form (GDS-15). Initially, a replicated cut off score of ≥ 5 for dPD and <5 for ndPD was applied. To better understand the results, we secondarily applied a more extreme group analysis with ≥ 9 for dPD and 0 for ndPD. White matter integrity between groups was compared between groups using tract-based spatial statistics.

**Results and Conclusion:** The current study did not reveal significant differences in white matter microstructure between dPD and ndPD groups at the whole brain level or in specific regions of interest. The extreme group results were consistent. These findings did not replicate previous work that found reduced white matter integrity in limbic prefrontal regions in dPD relative to ndPD. The current study highlights the need for more replications of neuroimaging research.

## Introduction

Parkinson's disease (PD) is a neurodegenerative movement disorder with comorbid depression in up to 35% of cases (1, 2). Despite the prevalence of PD related depression, research in this area has been minimal and inconclusive to date. As a result, depression in PD is less well understood and often misdiagnosed, contributing to a reduced quality of life for individuals with PD and depression, despite successful treatment for motor symptoms (1).

While assessment tools and treatments are available for PD depression, diagnosis remains complicated by several factors. Foremost, it is often difficult to separate depression symptoms from other PD symptoms (3, 4). For instance, patients may report feeling fatigued, less energetic, and having difficulty with initiating activity; yet, it is unclear whether these symptoms are a result of depression, motor impairment, or another cause entirely.

Recently, several neuroimaging studies have focused on PD depression with the goal of characterizing its underlying neuropathology. In particular, diffusion tensor imaging (DTI), which is able to capture microstructural characteristics of white matter, holds potential for detecting changes in the pathways affected in PD depression. The two most commonly used DTI indices are fractional anisotropy (FA), which measures directionality and is high in intact white matter, and mean diffusivity (MD), which measures diffusion in all directions and is low in intact white matter (5). Indices of axial (AD) and radial diffusivity (RD) provide additional indices that are thought to relate to axonal degeneration and demyelination when increased (5).

To date, although many studies have used DTI to study PD and depression separately, a limited number of studies have investigated depression in PD using DTI. Matsui et al. (6) compared depressed and non-depressed PD groups and found reduced white matter integrity in the anterior cingulate cortex (ACC), in the depressed PD group. Findings from Huang et al. (7) revealed reduced integrity in the frontal regions and connections to the subcortical-limbic regions, similar to other neuroimaging studies (8, 9). However, the most recent investigation found no statistically significant white matter integrity differences between PD individuals with and without depression, nor did FA and MD values correlate with symptoms of depression, contributing to mixed findings (10). Altogether, results across studies are conflicting and investigations of depression in PD need to be expanded upon and replicated.

## Present Study

The goal of the current study was to replicate a previous investigation of DTI metrics associated with depression in PD [see Huang et al. (7)]. The present study used data from the Parkinson's Progression Markers Initiative (PPMI) database to examine a larger sample size and more sensitive measures of DTI and depression, while using consistent parameters and statistical methods to previous research [as detailed in Huang et al. (7)].

Although the main aim was replication, a secondary objective of the current study was to extend previous research by applying more sensitive approaches: (1) by employing a region of interest based analysis to investigate the specific regions identified in Huang et al. (7), and (2) by restructuring the dPD and ndPD groups based on more extreme manifestations (or lack thereof) of depression.

Based on previous research, it was hypothesized that the dPD group would show reduced white matter integrity (as indicated by lower FA and higher MD/AD/RD) in fronto-subcortical limbic networks compared to the ndPD group and that these differences would become more widespread when groups were differentiated based on more extreme depression scores.

## Methods

Data were retrieved from PPMI, a public-private partnership funded by the Michael J. Fox Foundation for Parkinson's research, led by principle investigator Dr. Ken Marek. PPMI provides multifaceted data, including clinical, genetic, and neuroimaging tests, with the aim of uncovering various biomarkers useful for the early detection of Parkinson's disease (11). For more detail of specific study eligibility criteria and up-to-date information on the study, please see the PPMI website: www.ppmi-info.org. The University of Victoria Human Research Ethics Board approved the secondary data analyses for the current study.

All participants were diagnosed with Parkinson's disease (PD) by their own health care providers and diagnosis was confirmed by the primary PPMI investigator. *De novo* PD participants who received baseline testing on DTI and depression measures were eligible for inclusion.

## Measures

### Depression

Symptoms of depression were assessed on a well-validated measure in PD: the Geriatric Depression Scale short form (GDS-15). This self-report measure includes 15 yes/no questions, which were totaled for each participant. Scores on the GDS-15 determined the groups for comparison: initially, a non-depressed group (ndPD; <5 total GDS-15; *N* = 19) and a depressed group (dPD; ≥ 5 GDS-15; *N* = 18) and then, an extreme non-depressed group (exndPD; = 0 total GDS-15; *N* = 10) and an extreme depressed group (exdPD; ≥ 9 GDS-15; *N* = 10).

### Diffusion Tensor Imaging

All images were acquired with a Siemens 3T TIM Trio scanner with a 12 channel Matrix head coil. DTI data were acquired with a spin echo, echo planar imaging sequence with 64 gradient directions and a b-value of 1000 s/mm^2^, with a voxel size of 2 mm^3^.

### Image Preprocessing

Data preprocessing was carried out with FMRIB Software Library (FSL; version 5.0.10). Pre-processing steps included eddy current correction to correct for motion and artifacts (12), brain extraction to isolate brain tissues, and creation of individual binary brain masks [BET; Smith (13)], which were manually verified. Next, diffusion tensors were fit to every voxel using *DTIFIT*, outputting the FA, MD, AD, and RD values for each subject necessary for statistical comparison (14, 15).

### Tract-Based Spatial Statistics

Voxel-wise statistical analyses were performed using Tract-based Spatial Statistics (TBSS) in FSL (16–18) to compare dPD and ndPD groups as well as exdPD and exndPD groups. TBSS was used to create average tracts across subjects through registration of individual data onto a skeleton in 1 x 1 x 1 mm standard space (FMRIB59_FA). Then, mean FA/MD/AD/RD images were created and thresholded (FA > 0.3) to create the mean FA skeleton. Next, each participant's FA/MD/AD/RD data were projected onto the thresholded mean skeleton. Voxel-wise statistical analysis of the white matter skeleton was performed using Randomize, FSL's nonparametric permutation inference tool (5,000 permutations) with threshold free cluster enhancement to correct for multiple comparisons (*p* < 0.05). ROI analyses were carried out using the JHU ICBM-DTI-81 White-Matter labels and JHU White-Matter Tractography Atlases in *FEATQuery*, to assess the uncinate fasciculus, thalamic radiation, longitudinal fasciculus, and forceps minor, bilaterally.

### Behavioral

Groups were compared on several demographic and clinical measures using a two-way independent samples *t*-test in R Studio (RStudio version 1.0.153). Specifically, the depressed and non-depressed groups and the extreme depressed and extreme non-depressed groups were compared separately for differences in age, education level, Montreal Cognitive Assessment (MoCA) scores, Movement Disorder Society reviewed Unified Parkinson's Disease Rating Scale (MDS UPDRS)—III (movement scale) scores, Freezing of Gait scores, Hoehn and Yahr stages, duration of PD, and GDS-15 scores (see Table 1).

**Table 1:** Demographic data for depressed, non-depressed and extreme depressed, extreme non-depressed Parkinson's disease groups.

**Demographics**	**Depressed PD**	**Non-depressed PD**	***p*-value**	**Extreme depressed PD**	**Extreme non-depressed PD**	***p*-value**
	**(*n* = 18)**	**(*n* = 19)**		**(*n* = 10)**	**(*n* = 10)**	
Age	61.94 ± 8.48	62.00 ± 8.54	0.98	62.40 ± 12.57	61.40 ± 11.77	0.86
Education	15.22 ± 2.92	14.63 ± 3.22	0.56	15.40 ± 2.84	14.30 ± 2.71	0.39
Ethnicity	1 Hispanic; 1 African American; 2 Asians; 14 Caucasians	1 not specified; 18 Caucasians		1 African American; 9 Caucasians	10 Caucasians	
Males: females	11:7	11:8	0.85	6:4	6:4	1.00
MoCA	27.28 ± 1.81	27.73 ± 1.97	0.47	27.40 ± 2.17	28.2 ± 1.93	0.40
MDS UPDRS–III	27.78 ± 14.55	23.11 ± 10.25	0.26	22.20 ± 5.96	20.30 ± 7.85	0.55
Freezing of gait	0.06 ± 0.24	0.05 ± 0.23	0.97	0	0	–
Hoehn and Yahr	1.89 ± 0.47	1.74 ± 0.45	0.32	2.10 ± 0.32	1.70 ± 0.48	0.40*
PD duration	6.89 ± 0.96	6.74 ± 0.93	0.63	6.90 ± 0.99	6.30 ± 0.82	0.16
GDS-15	7.0 ± 2.45	1.95 ± 1.18	0.00*	11.13 ± 2.31	0.0 ± 0.0	0.00*
Anti-depressant medication	17 no, 1 yes	17 no, 2 yes		10 no, 0 yes	9 no, 1 yes	

Means and standard deviations or raw numbers shown.

* p < 0.05.

*MoCA, Montreal Cognitive Assessment; MDS UPDRS, Movement Disorder Society Unified Parkinson's Disease Rating Scale Part III (movement index); GDS-15, Geriatric Depression Scale short form. Colour to highlight and distinguish the 4 study groups*.

## Results

### Tract Based Spatial Statistics

A statistical comparison of FA, MD, AD, and RD values between the dPD and ndPD groups and the exPD and exndPD groups did not reveal statistically significant differences in white matter integrity at the whole brain level or in specific ROIs.

### Behavioral

There were no significant differences in age, sex, education level MoCA, UPDRS—III, Freezing of Gait, or duration of PD. There was a significant difference between the extreme groups in their Hoehn and Yahr stages, but not between the depressed and non-depressed groups. There was a significant difference between both sets of groups on the GDS-15 (see Table 1).

## Discussion

The aim of the current study was to determine if there are reliable microstructural differences in specific white matter tracts between individuals with dPD compared those with ndPD. In keeping with previous findings, it was hypothesized that there would be differences in white matter microstructure between the two PD groups associated with depression symptoms. Conversely, the current study's results revealed no significant differences in mean FA, MD, AD or RD values between the dPD and ndPD groups at a whole-brain or ROI level. Foremost, these findings do not support the replication of a recent investigation by Huang et al. (7) that compared DTI metrics of white matter integrity between PD groups with high and low symptoms of depression. However, the results of the current study did replicate the results of a more recent investigation of depression in PD, who likewise used PPMI data and comparable analysis techniques, and found no significant FA or MD white matter differences between a dPD and ndPD group (10). Overall, several studies using comparable methods to both the current study and Huang et al. (7) have found mixed results (6, 9, 10).

Importantly, this is the first study to our knowledge that has looked at white matter microstructure in the most severe symptoms of depression (i.e., extreme groups approach). Even with these more sensitive approaches, there were no significant differences found between the extreme dPD and ndPD groups. Previous studies that had not found differences between these groups interpreted it as a result of using mild depression scores to distinguish the depressed group (10). However, the extreme group analysis in the current study suggests that there are not significant differences even when individuals with clinically significant levels of depression are compared to individuals with no depression. These results suggest that there is not a neuropathological change to the integrity of white matter in individuals who experience depression in PD. However, it remains possible that there are changes to gray matter regions and that other approaches, such as voxel based morphometry may be more sensitive to the neuropathological changes associated with depression in PD.

### Replication: Challenges and Recommendations

Reliable microstructural white matter differences between dPD and ndPD groups were not found in the current study and studies to date remain inconclusive, with mixed findings. A first possible explanation for the mixed findings relates to variability in the assessment measures and diagnoses of depression. It is possible that participants in different studies had different severities and/or symptoms of depression that led to varying results.

Second, DTI acquisition parameters and statistical analytic approaches vary across DTI investigations of PD depression. MRI and DTI are promising tools for research and clinical diagnosis due to their non-invasive nature; however, there remains variability within acquisition parameters across studies. Acquisition parameters, including field strength (e.g., 1.5 vs. 3.0 Tesla) and number of gradient directions, influence the FA and MD values and thus reduce accuracy in comparison across studies that use different parameters (19). Guidelines for best practice in reproducible neuroimaging studies have been put forth and should be used to guide methodology of future DTI investigations (20, 21). Furthermore, correcting for multiple comparisons in voxel-wise statistical comparisons is especially important in analyses requiring a high number of voxel-based comparisons, like in TBSS, in order to reduce the risk of false discovery rates, or Type 1 error (20). Only two studies cited herein have reported whether or not their results were corrected for multiple comparisons (7, 10). Notably, although TBSS has gained popularity, perhaps as a result of its user-friendly framework, there have also been challenges the reliability of the technique, which has yet to be thoroughly established (18).

Third, the heterogeneous nature the PD diagnosis means no two PD samples are exactly alike. In addition to depression as a symptom of PD, other non-motor symptoms can be comorbid in PD, including anxiety, rapid eye movement (REM) sleep disorder, and olfactory impairments (3). In particular, controlling for often indiscernible neuropsychiatric comorbidities is a major challenge. As a result, comparisons across clinical PD populations is not always consistent.

A fourth factor influencing replication is sample size. DTI investigations, like other neuroimaging research, tend to be underpowered due to smaller sample sizes and lack of a priori effect size estimation. Small samples are highly typical of neuroimaging studies as a result of many factors, such as time and cost of imaging, preprocessing duration for each subject, and constraints from necessary control variables. Future research should aim to use the largest sample sizes available. Multi-site standardized and accessible datasets, such as the PPMI, will likely help with this goal as data continues to accumulate over time.

More generally, despite many studies advocating for the importance of methodological replication in validating neuropsychological science, a barrier is that only a small percentage of journals explicitly state whether they publish replication studies (21), and this remains a challenge for future research. If more journals explicitly stated their position on replication studies, and ideally encouraged them, this would likely motivate more replication studies and advance translational research, which requires reproducibility.

## Data Availability

The dataset is publicly available through the Parkinson's Progression Markers Initiative (ppmi-info.org/data).

## Author Contributions

CL and JG designed the study. CL and LO analyzed the data. CL, LO, and JG interpreted the results. CL wrote the initial draft of the manuscript with critical input from LO and JG.

### Conflict of Interest Statement

The authors declare that the research was conducted in the absence of any commercial or financial relationships that could be construed as a potential conflict of interest.
